# IGF2BP2 Promotes Epithelial to Mesenchymal Transition and Metastasis through Stabilizing HMGA1 mRNA in Gastric Cancer

**DOI:** 10.3390/cancers14215381

**Published:** 2022-10-31

**Authors:** Jun Ouyang, Junqing Li, Dongwei Li, Jianlong Jiang, Tengfei Hao, Yujian Xia, Xiaofang Lu, Changhua Zhang, Yulong He

**Affiliations:** 1 Department of Gastrointestinal Surgery, Dongguan Tungwah Hospital, Dongguan 523000, China; 2 Digestive Disease Center, The Seventh Affiliated Hospital, Sun Yat-Sen University, Shenzhen 518107, China; 3 Department of Pathology, The Seventh Affiliated Hospital, Sun Yat-Sen University, Shenzhen 518107, China

**Keywords:** gastric cancer, IGF2BP2, HMGA1, metastasis, EMT

## Abstract

**Simple Summary:**

Gastric cancer (GC) is one of the most common malignancies of the digestive system and ranks third among the causes of cancer-related death worldwide. Local deep invasion or distant metastasis in gastric cancer is usually associated with poor prognosis. Therefore, it is imperative to explore the mechanisms of GC metastasis. In this study, we identified that insulin-like growth factor 2 mRNA-binding protein 2 (IGF2BP2) overexpression in GC tissues was significantly associated with the deterioration of survival in GC patients. We then proved that IGF2BP2 accelerated the epithelial–mesenchymal transition (EMT) of GC by directly interacting with HMGA1 mRNA. Our findings suggest that IGF2BP2 may play an important role in the metastasis of GC.

**Abstract:**

As an RNA-binding protein, insulin-like growth factor 2 mRNA-binding protein 2 (IGF2BP2) is involved in enhancing the progression of a few malignant tumors by recognizing N6-methyladenosine on targeted RNA. However, the specific effects of IGF2BP2 on gastric cancer (GC) and the underlying mechanisms remain unclear. In this study, the expression level of IGF2BP2 was evaluated by analyzing data from a public database and performing immunohistochemical staining with GC specimens. The effect of IGF2BP2 on GC cell metastasis was investigated by Transwell assays and animal studies. RNA immunoprecipitation (RIP) was performed to identify potential mRNA bound to IGF2BP2. The levels of these identified RNAs were measured by RT-PCR, while corresponding proteins were quantified via Western blot. It was revealed that IGF2BP2 expression in GC tissues was significantly upregulated, and its overexpression was significantly associated with worse survival in GC patients. The aberrant expression of IGF2BP2 was demonstrated to promote the invasion and metastasis of GC cells by both in vivo and in vitro experiments. In subsequent experiments, it was then verified that by directly interacting with HMGA1 mRNA, IGF2BP2 augmented its stability and thus increased its expression. The knocking down of IGF2BP2 could significantly decrease the migration and invasion of GC cells, which could be reversed by increasing HMGA1 expression. Additionally, both in vitro and in vivo epithelial–mesenchymal transition (EMT) of GC cells were enhanced by IGF2BP2/HMGA1 axis. In conclusion, it was proven in our study that the IGF2BP2/HMGA1/EMT axis contributed to GC metastasis, suggesting its potential as a novel predictive and therapeutic biomarker for GC.

## 1. Introduction

As one of the most widespread and deadly cancers in the world, gastric cancer (GC) has some of the highest incidence (5.7%) and mortality (8.2%) rates [1]. Due to advances in diagnosis and comprehensive treatment, the survival rates were significantly enhanced in GC patients [2]. However, the prognosis for advanced GC patients is still rather poor, even after the application of targeted drugs or immunotherapy [3]. Therefore, the unraveling of the molecular mechanisms contributing to GC is a very important issue for us since acquaintance with these mechanisms will enable us to devise novel treatment strategies and, thus, improve the survival of GC patients.

Belonging to the insulin-like growth factor 2 (IGF-2) messenger RNA (mRNA)-binding protein family, IGF2BP2 is involved in modulating the localization, stability, and translation of bound mRNAs [4,5]. In some previous studies, IGF2BP2 was reported to encourage the progression of a few malignant tumors, such as breast tumors [6], non-small-cell lung tumors [7], and bowel cancer [8,9]. Furthermore, IGF2BP2 has been repeatedly reported by previously published studies to play roles as an N^6^-methyladenosine (m^6^A) reader [10]. In an IGF2BP2-dependent manner, METTL3 increases the progression of colorectal cancer [11]. In pancreatic cancer, IGF2BP2 facilitates cancer occurrence and promotes stem-cell-like properties by regulating DANCR as an m^6^A reader [12]. However, studies on the role of IGF2BP2 in GC are few. Su Y et al. suggested that the level of IGF2BP2 in GC tissues and the frequency of autoantibodies with IGF2BP2 in sera were separately associated with cell differentiation and tumor metastasis [13]. It was then shown by Liu X et al. that polymorphisms of IGF2BP2 might independently predict the responses of patients with metastatic GC to chemotherapy [14]. Additionally, it was demonstrated by Meistere et al. that, compared with healthy controls, IGF2BP2 predominantly reacted with the sera of GC patients [15]. Thus, given the conclusions of these studies, it could be inferred that IGF2BP2 might be associated with the occurrence and progression of GC. However, its specific effects on GC and the molecular mechanisms through which it contributes to GC need to be further elucidated.

High-mobility group A1 (HMGA1) is a member of nonhistone chromatin proteins and is widely present in eukaryotic cells [16]. HMGA1 regulates transcription by altering the structure of chromatin, thereby regulating a variety of biological events, such as proliferation, invasion, and metastasis [17]. Studies have shown that HMGA1 is upregulated in various tumors, including non-small-cell lung cancer, colon cancer, and breast cancer [18]. As an architectural transcriptional factor, HMGA1 binds to the promoters of the EMT-related genes E-cadherin, vimentin, and Twist1 and modulates their transcription [19]. In colorectal cancer, the LINC00460/DHX9/IGF2BP2 complex promotes proliferation and differentiation by stabilizing HMGA1 mRNA, depending on m^6^A modification [20]. HMGA1 is also upregulated in GC tissues and predicts poor prognosis; the overexpression of HMGA1 could improve the proliferation and migration abilities of GC cells [21,22]. However, its specific regulation mechanism is still unclear. 

As the initial stage of the carcinoma metastatic cascade, the activation of EMT can provide tumor cells with the ability to migrate and invade before they spread from the primary lesion into the circulation [23]. EMT is a highly complex and dynamic process in which the epithelial features of tumor cells (including stable epithelial cell-cell junctions, apical-basal polarity, and interactions with basement membrane) are suppressed by gene expression changes and post-translational regulatory mechanisms, with obtaining mesenchymal features [24]. E-Cadherin is essential for maintaining the epithelial phenotype. During the EMT process of GC, the E-cadherin expression of GC cells is lost, but abundant mesenchymal markers such as N-cadherin and Vimentin are expressed [25]. Furthermore, several key transcription factors (including Snail and ZEB) repress E-Cadherin expression through target E-box in the E-cadherin promoter [26]. EMT is a key progression step associated with poor prognosis of GC. Therefore, it is necessary to have a better understanding of the regulatory mechanisms of EMT during GC metastasis so as to promote the development of new therapeutic strategies.

In this project, by analyzing data from public databases and performing the immunohistochemical staining of clinical specimens, the level of IGF2BP2 was verified to be remarkably improved in gastric cancer tissues, and its overexpression predicted worse survival for GC patients. It was then demonstrated by functional analyses that IGF2BP2 promoted both in vivo and in vitro metastases of GC cells. Furthermore, it was verified through mechanistic studies that it was by directly interacting with HMGA1 mRNA and increasing its stability that IGF2BP2 facilitated the ability of epithelial-to-mesenchymal transition (EMT) in GC cell lines. Given these results of our study, IGF2BP2 could be a potential biomarker with prognostic and therapeutic value in GC.

## 2. Materials and Methods

### 2.1. Bioinformatics Analysis

The RNASeq data in GC were initially acquired from The Cancer Genome Atlas (TCGA) (https://cancergenome.nih.gov/ (accessed on 20 September 2019)). The effects of IGF2BP2 mRNA level on prognoses of GC patients were evaluated through Online Kaplan–Meier plotter (http://www.kmplot.com/ (accessed on 20 September 2019)). Next, we further evaluated the relationship between the mRNA level of IGF2BP2 and that of HMGA1 using Online LinkedOmics (http://www.linkedomics.org/ (accessed on 26 November 2019)) [27].

### 2.2. Clinical Samples

In total, 173 cases from 2004 to 2007 were randomly chosen among GC patients having undergone curative surgery at The First Affiliated Hospital, Sun Yat-sen University (FAHSYSU). Clinical follow-ups of these 173 patients were terminated on 30 June 2018. These 173 GC patients did not receive neoadjuvant therapies. The staging of tumors in these 173 patients was reassessed based on the eighth version of the American Joint Cancer Committee (AJCC) TNM classifications. Informed consent in written form was acquired from each individual inpatient. Consent to conduct the present research was granted by the Ethics Committee of FAHSYSU.

### 2.3. Cell Lines and Cell Culture

GC cell lines, such as AGS, SGC7901, and MKN28, studied in this research were obtained from the Shanghai Institute of Cell Biology, Chinese Academy of Sciences (Shanghai, China). AGS cells were kept in DMEM/F-12 (Gibco, Logan, UT, USA), while SGC7901 and MKN28 were maintained with RPMI 1640 Medium (Gibco) containing 10% FBS (Gibco) at a temperature of 37 °C and in an atmosphere of 5% CO_2_.

### 2.4. Transfection and Stable Cell Lines Construction

Through synthesizing and cloning IGF2BP2 ORF and lentiviral negative control sequence into the lentiviral plasmid pEZ-Lv201 (Genecopeia, Guangzhou, China), we established stable IGF2BP2 overexpression, and control cell lines were called EX-H1849-Lv201 and EX-NEG-Lv201, respectively, which were then transfected into AGS and SGC7901 cells. Seventy-two hours later, stable cell lines were constructed over 4 weeks; the cells were cultured in a puromycin-added medium. In order to establish IGF2BP2-knockdown cell lines, we cloned oligonucleotides encoding short hairpin RNA (shRNA) into lentiviral vector psi-LVRU6GP (Genecopeia), which were named sh-1 and sh-2, respectively. The negative control utilized in this study was a scrambled shRNA named sh-Ctrl. Sequences of sh-1, sh-2, and sh-Ctrl were as follows: GGAAGTTGATTACTCAGTCTC, GGACCAAGATAACAATCTCAT, and ACAGAAGCGATTGTTGATC. After being transfected with sh-RNAs (sh-1, sh-2, or sh-Ctrl), stable cell lines were established over 4 weeks; GC cells were cultured in a puromycin-supplemented medium (2 μg/mL puromycin). To construct GC cells in which HMGA1 was overexpressed, we then transfected HMGA1-overexpression-promoting plasmid (GV-HMGA1) and negative-control plasmid (GV-Vector) into GC cells with Lipofectamine 3000 (Invitrogen, Carlsbad, CA, USA), while the GV492 used as the carrier was devised and completed by GeneChem (Shanghai, China).

### 2.5. RNA Extraction and qRT-PCR

Using AG RNAex Pro Reagent (Accurate Biology, Changsha, China) and Evo M-MLV RT Premix Kit (Accurate Biology), the total RNA was extracted following the protocol obtained from the manufacturer and was reverse-transcribed into cDNA subsequently. Following the manufacturer’s protocols, quantitative real-time PCR (qRT-PCR) assays were performed on CFX96 Touch Deep Well Real-Time PCR System (Bio-Rad, Guangzhou, China), with SYBR^®^ Green Premix Pro Taq HS qPCR Kit (Accurate Biology). The primers were as follows: GAPDH, forward sequence 5′-ACAACTTTGGTATCGTGGAAG-3′ and reverse sequence 5′-GCCATCACGCCACAGTTTC-3′; IGF2BP2, forward sequence 5′-AGCTAAGCGGGCAT-3′ and reverse sequence 5′-CCGCAGCGGGAAATCAATCT-3′; and HMGA1 forward sequence 5′-AGCGAAGTGCCAACACCTA-3′ and reverse sequence 5′-TGGTGGTTTTCCGGGTCTTG-3′.

### 2.6. Western Blot

GC cell lines were lysed by RIPA, a buffer incorporating phosphatase and protease inhibitor reagents (Beyotime Biotechnology, Shanghai, China), for extracting total protein, which was then measured by BCA assay. Proteins were shifted into PVDF membranes (Merck Millipore, Billerica, MA, USA) after the same amount of these resolved in sodium dodecyl sulfate-polyacrylamide gel electrophoresis (SDS-PAGE). Subsequently, these PVDF membranes were incubated in a 5% bovine serum albumin (BSA) solution at about 25 °C for more than 1 h. Next, these membranes were immunoblotted with each primary antibody at a temperature of 4 °C for one night. The next day, the PVDF membranes were washed three times before incubation in a buffer including HRP-conjugated affinipure goat anti-rabbit IgG (SA00001-2, Proteintech, Wuhan, China) at about 25 °C for more than 1 h. Next, these PVDF membranes covered with antibody-conjugated protein bands were washed with TBST buffer for at least 20 min, which was followed by detection with a BeyoECL Plus Kit (Beyotime Biotechnology). Antibodies such as anti-GADPH (10494-1-AP), anti-IGF2BP2 antibody (11601-1-AP), anti-ZEB1 antibody (21544-1-AP), anti-Vimentin antibody (10366-1-AP), anti-SNAI1 antibody (13099-1-AP) and anti-N-cadherin antibody (22018-1-AP), as well as an anti-E-cadherin antibody (20874-1-AP), were obtained from Proteintech, while anti-HMGA1 antibody (AF5218) was produced by Affinity Biosciences (Changzhou, China).

The expression level of GADPH was used as the basis of calculation.

### 2.7. Cell-Function Assays

Transwell assays were designed to determine the influence of IGF2BP2 on the migration and invasion in GC cell lines. Regarding migration assay, on the upper chamber, 5 × 10^4^ cells were cultured in serum-free medium (8-μm pore size, Corning, NY, USA), while there was a 10% FBS-contained medium in the lower chamber. For invasion assay, the same amounts of cells were cultured on the upper insert covered with 10% Matrigel (Corning). The incubation time for SGC7901 and MKN28 cells was 48 h, but 24 h for AGS cells. After incubation, cells remaining in the upper chamber were wiped off using swabs. Next, cells were fixed with 4% paraformaldehyde and dyed with 0.1% crystal violet after passing through the membrane. Five different fields of view under the microscope were used to count the cells that migrated into or invaded the lower chamber. All cell-function experiments in this study were repeated at least three times.

### 2.8. RNA Immunoprecipitation

According to the protocol provided by the manufacturer, EZ-Magna RIP RNA Binding Protein Immunoprecipitation Kit (17-700, Millipore) was used to perform RNA immunoprecipitation (RIP) experiments. RNA binding proteins for IGF2BP2 were immunoprecipitated using anti-IGF2BP2 antibody and IgG. To quantify the enrichment of RNA bound to IGF2BP2, we then performed RT-PCR analyses with these identified RNAs as substrates, the products of which were then detected through 3% agarose gel electrophoresis.

### 2.9. RNA Stability Assays

GC cell lines were processed using 2.5 μg/mL actinomycin D (MedChemExpress, Shanghai, China) for 0, 8, and 16 h, respectively. Next, total RNA was isolated from these actinomycin-D-processed cells with AG RNAex Pro Reagent and analyzed by RT-PCR with GAPDH as the normalization. Finally, the half-lives of mRNA were detected by linear regression analysis.

### 2.10. Animal Studies

Five-week-old female BALB/c nude mice were used to construct an animal model to evaluate the effects of IGF2BP2 expression on GC in vivo. For an in vivo metastasis model, 2 × 10^6^ SGC7901 cells in which IGF2BP2 were stably overexpressed or control SGC7901 cell lines were injected into the tail vein. After 7 weeks, all these mice were killed, and metastatic lung lesions were removed and examined by immunohistochemical (IHC) and HE staining. All these assays were permitted and supervised through the Experimental Animal Ethics Committee of Ruiye Model Animal (Guangzhou, China) Biotechnology Co., Ltd. (approval no. 20201010001).

### 2.11. Immunohistochemical Staining

Paraffin-embedded specimens of 173 GC patients were retrieved from the Department of Pathology, FAHSYSU. All animal specimens were immediately fixed with 4% paraformaldehyde and embedded in paraffin. According to the protocols provided by the manufacturer, we performed IHC staining utilizing these fixed and paraffin-embedded tissues with an Immunohistochemistry Kit (Sangon Biotech, Shanghai, China). Expression levels of IGF2BP2 in these tissues were determined in a semiquantitative manner [12]. The total score of each patient was measured by multiplying the staining intensity and staining area. Staining-intensity score in this study was defined based on the following criteria: 0 (negative staining), 1 (weak staining), 2 (moderate staining), and 3 (strong staining), while the score for staining area was calculated as follows: 0 (0%), 1 (1–10%), 2 (10–50%), and 3 (51–100%).

### 2.12. Statistical Analysis

For continuous variables, the comparisons were accomplished by student’s *t*-test and one-way ANOVA, and categorical instances were analyzed by Fisher’s exact test and Chi-square test. Kaplan–Meier curves of the included patients were constructed and compared by log-rank test. The hazard ratio (HR) and 95% confidential interval (CI) were calculated by Cox regression analysis. All the statistical analyses included in this research were performed by SPSS (version 17.0, IBM, Chicago, IL, USA), and a *p*-value was considered statistically significant when less than 0.05.

## 3. Results

### 3.1. Increased Expression of IGF2BP2 Was Significantly Correlated with Worse Survival in GC Patients

**Figure 1:**
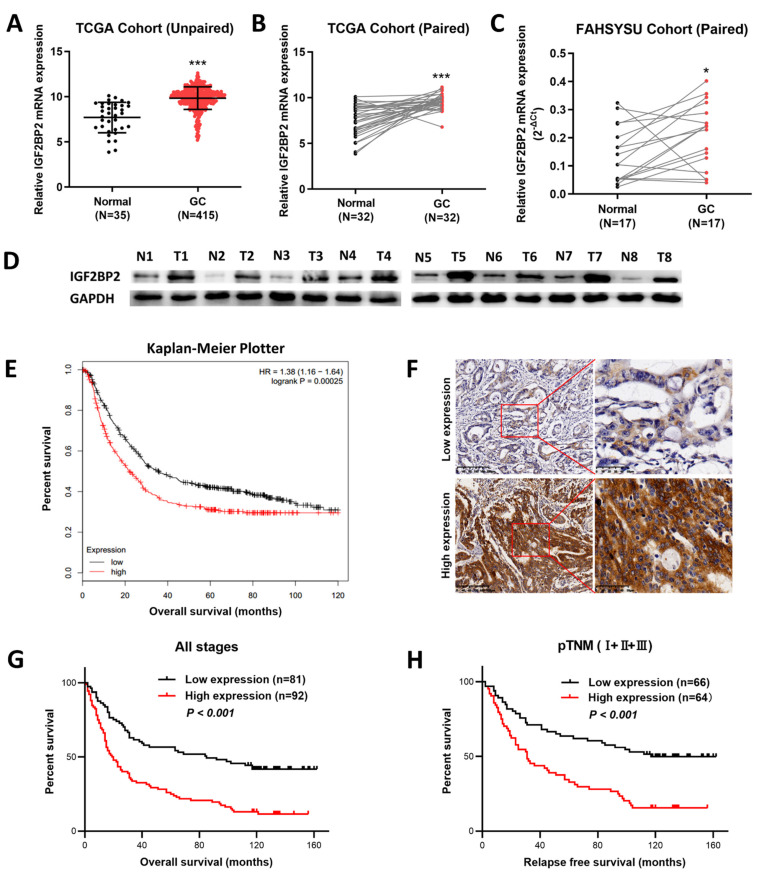
The expression of IGF2BP2 in GC tissues is remarkably upregulated, which predicts poor survival in GC patients. (**A**,**B**) The data from the TCGA database show the expression levels of IGF2BP2 in unpaired or paired GC and normal gastric tissues. (**C**) Expression levels of IGF2BP2 in paired GC and normal gastric tissues detected RT-PCR. (**D**) Representative results of the IGF2BP2 protein upregulation in GC specimens determined by Western blotting. (**E**) The correlation of high or low IGF2BP2 expression with the overall survival of GC patients according to the Kaplan–Meier plotter database. (**F**) Representative results of immunohistochemical staining of IGF2BP2 in GC specimens (200×). (**G**,**H**) The expression of IGF2BP2 upregulation was obviously related to poor overall survival and relapse-free survival in GC patients through Kaplan–Meier survival analysis. * *p* < 0.05, *** *p* < 0.001. Molecular weights for proteins are shown in the uncropped Western blot images (Figure S1).

Firstly, the expressions of IGF2BP2 in the GC tissues were evaluated by analyzing relevant data from TCGA, revealing that compared to normal gastric tissues, the expression levels of IGF2BP2 in GC tissues were significantly higher (Figure 1A,B). Secondly, in order to affirm the higher expression levels of IGF2BP2 in the GC tissues, the RT-PCR and Western blotting assays were performed using paired GC specimens obtained from FAHSYSU, revealing that in comparison with that in adjacent gastric tissues, the expression of IGF2BP2 was significantly enhanced at both mRNA (Figure 1C) and protein (Figure 1D) level. Thirdly, with the aim of further assessing the prognostic significance of IGF2BP2 overexpression, the data from the TCGA database were analyzed using an online Kaplan–Meier plotter. The results showed that the higher the mRNA level of the IGF2BP2, the worse the overall survival (OS) of the patients (Figure 1E). Fourthly, the Kaplan–Meier analysis was used to evaluate the relationship between the protein expression of IGF2BP2 and the patients by IHC staining. Based on the IHC staining of 173 tissues, we found that the IGF2BP2 protein was mainly located in the cytoplasm. (Figure 1F). In this study, we also revealed that elevated IGF2BP2 expression was significantly related to tumor size, TNM stage, lymph node metastasis, and depth of invasion (Table 1). Additionally, the patients with high expression levels of IGF2BP2 had worse OS and relapse-free survival (RFS), as revealed by the Kaplan–Meier curves in Figure 1G,H. The univariate and multivariate Cox regression analyses also proved that a high expression level of IGF2BP2 is an independent predictor of OS and RFS, as demonstrated in Table 2 and Table 3, respectively. Combining the above results, we can conclude that the expression of IGF2BP2 in GC was significantly elevated and might be valued as a novel prognostic biomarker for patients diagnosed with GC.

### 3.2. In Vitro and In Vivo Metastasis of GC Is Promoted by IGF2BP2

**Figure 2:**
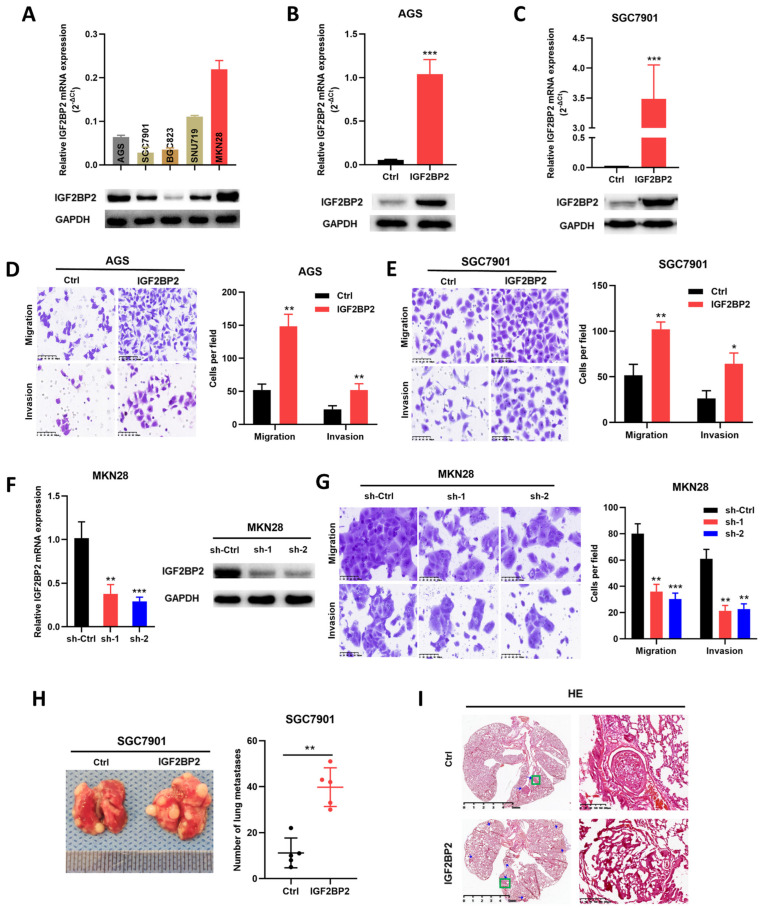
IGF2BP2 promotes in vivo and in vitro metastasis of GC cells. (**A**) The mRNA and protein levels of IGF2BP2 in GC cell lines. (**B**,**C**) Western blotting and RT-PCR were performed to confirm the stable IGF2BP2-overexpressing AGS and SGC7901 cells were constructed successfully. (**D**,**E**) The migration and invasion capability of AGS and SGC7901 cells were significantly increased by the upregulation of IGF2BP2. (**F**) Western blotting and RT-PCR were performed to confirm the stable IGF2BP2 knockdown MKN28 cells were constructed successfully. (**G**) Migration and invasion capabilities of MKN28 were significantly inhibited by the knocking down of IGF2BP2. (**H**) Representative images and quantification of metastatic lung tumors after IGF2BP2-overexpression or control SGC7901 cells were injected. (**I**) Representative images depicting lung-tumor l metastasis through HE staining. * *p* < 0.05, ** *p* < 0.01, *** *p* < 0.001. Molecular weights for proteins are shown in the uncropped Western blot images (Figures S2–S5).

In order to clarify/verify the biological functions of IGF2BP2 in GC, we subsequently established stable IGF2BP2-overexpressing GC cells (AGS and SGC7901) (Figure 2A–C). It was proven that both the migration and the invasion of AGS (Figure 2D) and SGC7901 (Figure 2E) were significantly enhanced by the upregulation of IGF2BP2 in Transwell assays. In addition to establishing stable IGF2BP2-overexpressing GC cells, we also established stable IGF2BP2 knockdown cell lines (Figure 2F). As expected, the knockdown of IGF2BP2 inhibited the migration and invasion of MKN28 (Figure 2G). Next, the effects of the IGF2BP2 expression level on the metastasis of GC cells in vivo were further assessed by animal experiments. IGF2BP2-overexpressing and corresponding control SGC7901 cells were injected through the tail vein. After being raised for seven weeks, the mice were euthanized, and lung tissues were collected, revealing that in comparison with the control groups, the number and size of both surface (Figure 2H) and coronal plane (Figure 2I) metastatic lesions were significantly obvious, which proved that the upregulation of IGF2BP2 significantly enhanced the lung metastasis of GC. Taking these results together, we drew the conclusion that IGF2BP2 played a critical role in promoting metastasis in GC.

### 3.3. IGF2BP2 Enhances HMGA1 mRNA Stability through Direct Interaction

**Figure 3:**
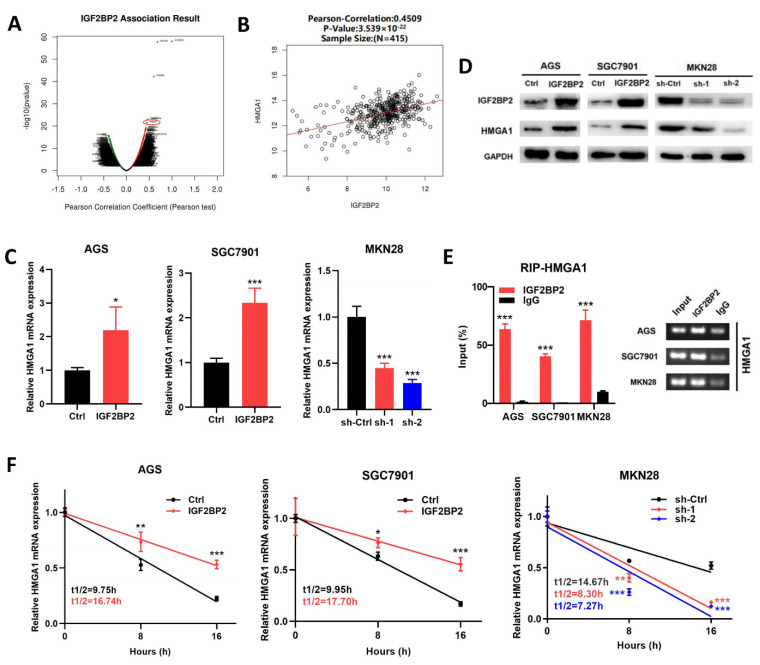
IGF2BP2 interacts with HMGA1 mRNA directly and enhances its stability. (**A**) Volcano plot illustrating the correlation between IGF2BP2 and other genes according to LinkedOmics database. (**B**) Correlation analysis of IGF2BP2 and HMGA1 according to LinkedOmics database. (**C**,**D**) The mRNA and protein levels of HMGA1 were upregulated by IGF2BP2 overexpression and downregulated by the knocking down of IGF2BP2. (**E**) It was proven by agarose electrophoresis and RT-PCR analysis of RIP assays that direct interactions took place between IGF2BP2 and HMGA1 mRNA in GC cells. (**F**) After actinomycin D (2.5 μg/mL) treatment of AGS, SGC7901, and MKN28 cells, the decay rates of mRNA of HMGA1 at certain times were analyzed by RT-PCR. * *p* < 0.05, ** *p* < 0.01, *** *p* < 0.001. Molecular weights for proteins are shown in the uncropped Western blot images (Figures S6–S8).

To explore the molecular mechanisms through which IGF2BP2 promoted the metastasis of GC, we initially analyzed the genes that were co-expressed with IGF2BP2 by LinkedOmics. As shown in Figure 3A, many genes were demonstrated to be significantly associated with IGF2BP2 expression, and HMGA1 was found to be positively associated with IGF2BP2 expression, suggesting the potential positive regulatory mechanism of IGF2BP2 (Figure 3B).

In order to testify that the expression level of HMGA1 was regulated by IGF2BP2, we then performed RT-PCR and Western blotting analyses with MKN28, SGC7901, and AGS cells. The results showed that the overexpression of IGF2BP2 increased HMGA1 expression at mRNA and protein levels, while the downregulation of IGF2BP2 resulted in a decrease in the mRNA and protein level of HMGA1 at the same time (Figure 3C,D). As an RNA-binding protein, IGF2BP2 performs its biological functions by binding to targeted RNAs [4]. As an open-source online instrument studying the interactions of RBP-mRNA from CLIP-seq, we identified HMGA1 mRNA as a potential target of IGF2BP2 by ENCORI (http://starbase.sysu.edu.cn/ (accessed on 26 November 2019)). RIP experiments were subsequently performed to test the enrichment between HMGA1 mRNA and IGF2BP2. It was revealed through the RIP assays performed with MKN28, SGC7901, and AGS cells that, compared with the enrichments of the HMGA1 mRNA precipitated by the control IgG, those precipitated by the anti-IGF2BP2 antibodies were significantly increased (Figure 3E), suggesting that the IGF2BP2 directly interacted with the HMGA1 mRNA. Furthermore, the RNA decay rates were assessed in both IGF2BP2-overexpressing and IGF2BP2-knockdownGC cells, which were compared with those in the corresponding control cells. It was demonstrated through the assessment of the RNA decay rate that in the SGC7901 and AGS cells, the IGF2BP2 overexpression led to consistently and significantly lengthened half-lives of HMGA1 mRNA (Figure 3F). By contrast, in the MNK28 GC cells, knocking down IGF2BP2 resulted in a significantly shortened, relatively approximate half-life of HMGA1 mRNA (Figure 3F). Considering these results, we concluded that the direct interaction between HMGA1 mRNA and IGF2BP2 maintained its stability and increased its expression.

### 3.4. IGF2BP2/HMGA1 Axis Enhanced the Migration and Invasion Ability in GC Cells

**Figure 4:**
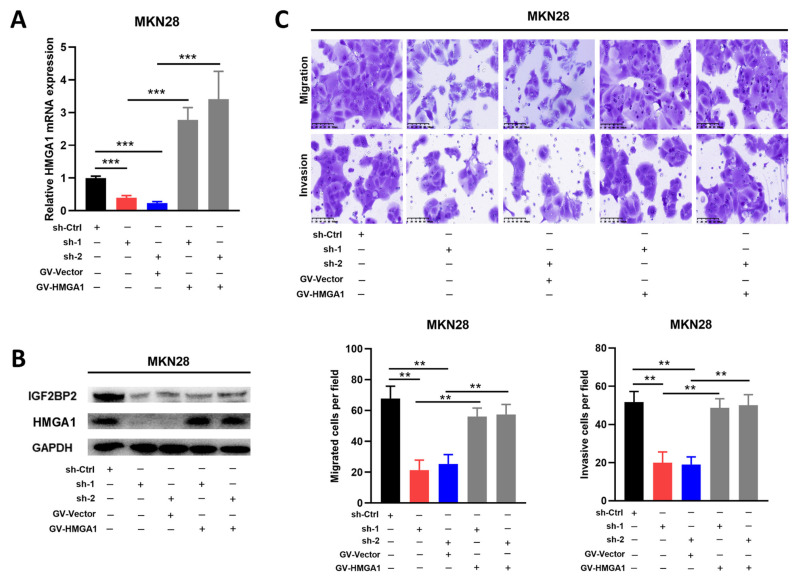
IGF2BP2/HMGA1 axis encourages migration and invasion capability in GC cell lines. (**A**,**B**) HMGA1 overexpression in MKN28 cells, in which IGF2BP2 knockdown was confirmed by Western blotting and RT-PCT analysis. (**C**) The migration and invasion capability in MKN28 GC cells reduced by knocking down of IGF2BP2 were rescued by HMGA1 overexpression. ** *p* < 0.01, *** *p* < 0.001. Molecular weights for proteins are shown in the uncropped Western blot images (Figure S9).

Despite the fact that IGF2BP2 plays a vital role in encouraging metastasis in GC, it is still unclear whether the IGF2BP2/HMGA1 axis is responsible. As mentioned above, we established stable IGF2BP2 knockdown and control MKN28 cells, into which the HMGA1 overexpression (GV-HMGA1) or control plasmid (GV-Vector) were transfected. The successful overexpression of HMGA1 in the IGF2BP2-knockdown MKN28 was confirmed through both qRT-PCR and Western blotting assays (Figure 4A,B). The decreased migration and invasion capability caused by the knockdown of IGF2BP2 was reversed by the ectopic expression of HMGA1 (Figure 4C). Considering all these findings, we drew the conclusion that the IGF2BP2/HMGA1 axis was responsible for encouraging the migration and invasion of the GC cells.

### 3.5. IGF2BP2/HMGA1 Axis Promotes EMT of GC

**Figure 5:**
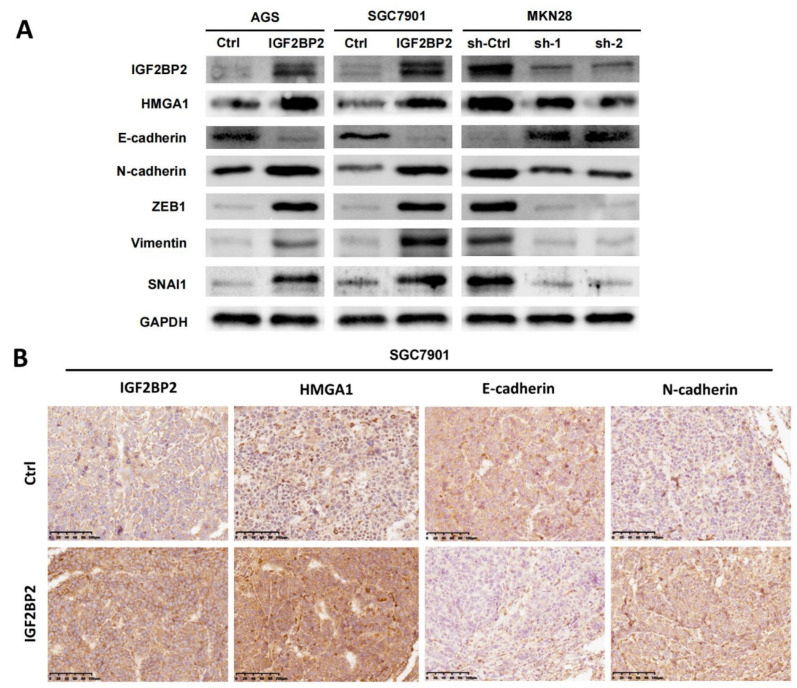
IGF2BP2/HMGA1 axis promotes EMT of GC. (**A**) The amounts of HMGA1, E-cadherin, N-cadherin, ZEB1, Vimentin, and SNAI1 and in AGS, SGC7901, and MKN28 cells in which IGF2BP2 was overexpressed or knocked down were evaluated by Western blotting analyses. (**B**) Immunohistochemical staining was completed to evaluate the amounts of HMGA1, E-cadherin, and N-cadherin in metastatic lung lesions removed from mice injected with IGF2BP2-overexpressing or control SGC7901 cells. Molecular weights for proteins are shown in the uncropped Western blot images (Figure S10).

Evidence supporting the conclusion that HMGA1 promoted the progression of GC by regulating EMT is continuously emerging [21]. Therefore, we further explored whether the IGF2BP2/HMGA1 axis regulated EMT in GC. It was demonstrated by Western blotting analysis that in IGF2BP2-overexpressing AGS and SGC7901 cells, the increased expression level of HMGA1 resulted in the upregulation of N-cadherin, ZEB1, Vimentin, SNAI1, and the low expression of E-cadherin (Figure 5A). By contrast, in IGF2BP2-knockdown MKN28 cells, decreased HMGA1 was related to the downregulation of N-cadherin, ZEB1, Vimentin, SNAI1, and the higher expression of E-cadherin (Figure 5A). A similar result to that was observed in the GC cell lines was discovered in the lung-metastatic model. It was revealed by the immunohistochemical staining of the IGF2BP2-overexpressing or control lung-metastatic lesions that the increased expression of HMGA1 was related to the reduced expression of E-cadherin and the upregulation of N-cadherin (Figure 5B). All these findings suggested that EMT’s mediation by IGF2BP2 was attributable to the overexpression of HMGA1.

## 4. Discussion

IGF2BP2 has been reported by multiple previous studies to be related to the development and metastasis of some cancers [4]. Furthermore, IGF2BP2 has been shown to be associated with the metastasis of GC [13]. However, its specific functions in GC and the molecular mechanisms through which it contributes to GC progression were not previously explored. In this research, it was clarified that IGF2BP2 was significantly upregulated in GC and that its overexpression was related to poor survival in GC patients. Furthermore, IGF2BP2 was proven by both in vivo and in vitro assays to encourage GC metastasis. It was then demonstrated by subsequent experiments that IGF2BP2 encouraged GC metastasis by directly interacting with HMGA1 mRNA and increasing its expression, as did the increase in EMT by the IGF2BP2/HMGA1 axis.

It has been reported that IGF2BP2 is associated with the development of glioblastoma [28], colorectal cancer [8], breast cancer [6], non-small-cell lung cancer [7], and pancreatic cancer [29]. However, studies on the roles of IGF2BP2 in GC are still scarce. Firstly, the data extracted from TCGA were analyzed, indicating that in comparison with normal gastric tissues, the expression levels of IGF2BP2 in GC were obviously upregulated, which matched the results of the RT-PCR and Western blotting analyses performed using the specimens from our center. Secondly, the prognostic significance of the IGF2BP2 expression level was evaluated by Kaplan–Meier analysis (online KaplanπMeier plotter), indicating that high expressions of IGF2BP2 resulted in worse OS in GC patients. The prognostic significance of IGF2BP2 expression was also evaluated by performing IHC staining with the 173 GC specimens and, subsequently, Kaplan–Meier analysis, demonstrating that the upregulated expression of IGF2BP2 was significantly related to worse OS and RFS. It was then proven that high IGF2BP2 expression was an independent prognostic factor for both OS and RFS through Cox regression analysis. Combining these findings, we inferred that elevated IGF2BP2 expression could predict poor prognosis in patients with gastric cancer.

Metastasis is the leading factor contributing to poor long-term outcomes of patients diagnosed with cancers [30]. IGF2BP2 was reported by a previously published study to encourage the metastasis of colorectal cancer [11]. However, whether IGF2BP2 promotes GC metastasis remained ambiguous. Therefore, the effects of IGF2BP2 on GC metastasis were subsequently evaluated in vivo and in vitro. It was demonstrated through Transwell experiments that the upregulation of IGF2BP2 significantly increased the migration and invasion capabilities in the AGS and SGC7901 cell lines, while the capabilities were significantly weak in MNK28 after IGF2BP2 was down-regulated. Next, the in vivo effects of IGF2BP2 on GC metastasis were assessed by animal experiments, revealing that the upregulation of IGF2BP2 significantly facilitated lung metastasis in SGC7901 cells. Taking all these results into consideration, we concluded that aberrant IGF2BP2 expression could encourage GC metastasis.

As an RNA binding protein, IGF2BP2 performs biological functions by directly binding to the target RNA [4], such as IGF2 [31], LAMB2 [32], and UCP1 [33]. In order to identify the target mRNAs of IGF2BP2 in GC, we first determined genes that were associated with IGF2BP2 expression via LinkedOmics, revealing that HMGA1 was positively associated with IGF2BP2. Subsequently, it was demonstrated that in AGS, SGC7901, and MKN28, HMGA1 expression was positively regulated by IGF2BP2 through RT-PCR and Western blotting analyses. Next, through ENCORI, we demonstrated that HMGA1 was a potential target of IGF2BP2. In a study by Dai et al., it was proven that IGF2BP2 is bound to HMGA1 mRNA in MEFs and RD cells [34]. However, whether the interaction between IGF2BP2 and mRNA described by Dai et al. was involved in GC was still unknown. In our study, the results of the RIP assays revealed that the direct interaction between HMGA1 mRNA and IGF2BP2 was involved in GC. Next, we evaluated the effect of IGF2BP2 on the stability of HMGA1 mRNA by RNA decay assays. We found that IGF2BP2 sustained the HMGA1 mRNA stability and prevented it from being degraded. IGF2BP2 was confirmed as an m^6^A reader in a study by H. Huang et al. [10]. Similarly, as an m^6^A reader, IGF2BP2 promoted stemness-like characteristics and pathogenesis in pancreatic cancer by stabilizing DANCR mRNA [12]. The study on colorectal cancer revealed that LncRNA LINRIS enhanced the stability of IGF2BP2, which increased the binding of IGF2BP2 to m^6^A-modified MYC [35]. Recent studies showed that IGF2BP2 promotes GC progression by targeting IGF1R and SIRT1 mRNAs and found that there are m^6^A modification sites on IGF1R and SIRT1 genes through bioinformatics analysis, so it is speculated that this promotion of IGF2BP2 may be mediated through m^6^A modification [36,37]. However, it is still unknown whether an m^6^A modification on HMGA1 mRNA was absolutely necessary for IGF2BP2 to regulate its expression. It was demonstrated by analyzing the m^6^A-sequencing data from METTL3 knockdown and control AGS cells that HMGA1 did not belong to the differentially expressed genes modified by m^6^A. This was also reported in a study by Yue et al. [25], suggesting that IGF2BP2 might modulate HMGA1 expression in a manner independent of m^6^A modification. The roles played by IGF2BP2 that are dependent on m^6^A modification in GC still need to be validated by more studies.

To confirm that IGF2BP2′s role in encouraging GC metastasis is attributable to the IGF2BP2/HMGA1 axis, we then performed rescue assays, the results of which demonstrated that IGF2BP2 promoted GC metastasis by increasing HMGA1 expression. As is commonly known, EMT is responsible for the initial stage of cancer metastasis [38]. Furthermore, HMGA1 could promote GC progression through EMT [21]. Therefore, we hypothesized that IGF2BP2/HMGA1 axis promoted EMT in GC. This was confirmed by subsequent experiments. It was revealed in Western blotting analyses that in IGF2BP2-overexpressing AGS and SGC7901 cells, the upregulation of HMGA1 led to the increased expression of N-cadherin, ZEB1, Vimentin, SNAI1, and the low expression of E-cadherin; the opposite was observed in the MKN28 cell lines when IGF2BP2 was knocked down. Similar effects were confirmed by the IHC staining of lung-metastatic lesions. These results suggested that IGF2BP2 accelerated EMT in GC by increasing the expression of HMGA1.

## 5. Conclusions

In conclusion, our findings reveal that IGF2BP2 accelerates EMT and metastasis in GC by increasing HMGA1 mRNA stability. Furthermore, the expression level of IGF2BP2 is distinctly higher in GC tissues and is correlated with poor survival in GC patients, showing its value as a new prognostic and therapeutic marker for GC.

## Figures and Tables

**Table 1:** Correlations between IGF2BP2 expression and clinicopathologic parameters of 173 GC patients.

Characteristics	No.	IGF2BP2 Expression		χ^2^	*p*	ρ ^†^	*p*
		Low (N = 81)	High (N = 92)				
Age		52.44 ± 11.98	58.38 ± 12.57				
<60 y	103	60	43	13.36	0.000 ***	0.278	0.000 ***
≥60 y	70	21	49				
Gender							
Male	116	54	62	0.01	0.919	−0.008	0.920
Female	57	27	30				
Tumor location							
Proximal	41	17	24	1.843	0.606	0.003	0.967
Middle	41	22	19				
Distal	75	36	39				
More than 2	16	6	10				
Tumor size							
<5 cm	71	40	31	4.381	0.036	0.159	0.037 *
≥5 cm	102	41	61				
Histologic type							
Adenocarcinoma	151	71	80	1.410	0.842	0.014	0.859
Squamous	1	1	0				
Signet ring	11	5	6				
Mucinous	7	3	4				
Undifferentiated	3	1	2				
Borrmann classification							
Ⅰ	12	6	6	6.169	0.104	0.170	0.026 *
Ⅱ	40	24	16				
Ⅲ	102	46	56				
Ⅳ	19	5	14				
Differentiation							
High	2	1	1	0.621	0.733	−0.056	0.464
Moderate	41	17	24				
Poor	130	63	67				
Depth of invasion							
T1	19	13	6	5.283	0.152	0.160	0.035 *
T2	21	11	10				
T3	98	44	54				
T4	35	13	22				
Lymph-node metastasis							
N0	48	31	17	8.533	0.036 *	0.191	0.012 *
N1	73	30	43				
N2	32	12	20				
N3	20	8	12				
Distant metastases							
M0	131	66	65	2.748	0.097	0.126	0.098
M1	42	15	27				
AJCC pTNM							
Ⅰ	33	22	11	7.451	0.059	0.184	0.015 *
Ⅱ	62	28	34				
Ⅲ	36	16	20				
Ⅳ	42	15	27				

ρ ^†^, Spearman’s rank correlation coefficient. * *p* < 0.05, *** *p* < 0.001.

**Table 2:** Overall survival analyzed through Cox proportional-hazard regression.

Characteristic	Univariate Analysis				Multivariate Analysis			
	*p*	HR	95.0% CI for Exp (B)		*p*	HR	95.0% CI for Exp (B)	
			Lower	Upper			Lower	Upper
Gender	0.025 *	1.512	1.054	2.168	0.020 *	1.571	1.073	2.300
Age	0.399	1.163	0.820	1.649				
Tumor location	0.662	1.046	0.855	1.280				
Tumor size	0.009 **	1.623	1.127	2.339				
Histologic type	0.423	1.069	0.908	1.258				
Borrmann classification	1.89 × 10^−4^ ***	1.680	1.275	2.213				
Differentiation	0.662	1.087	0.747	1.582				
Depth of invasion	5.46 × 10^−6^ ***	1.633	1.314	2.029				
Lymph-node metastasis	5.67 × 10^−9^ ***	1.677	1.404	2.003				
Distant metastases	4.58 × 10^−15^ ***	4.776	3.188	7.156	0.015 *	3.051	1.247	7.462
AJCC pTNM	2.05 × 10^−14^ ***	2.051	1.699	2.477				
IGF2BP2 expression	1.46 × 10^−6^ ***	2.406	1.672	3.462	1.90 × 10^−4^ ***	2.155	1.466	3.169

* *p* < 0.05, ** *p* < 0.01, *** *p* < 0.001.

**Table 3:** Relapse-free survival analyzed using Cox proportional-hazard regression.

Characteristic	Univariate Analysis				Multivariate Analysis			
	*p*	HR	95.0% CI for Exp (B)		*p*	HR	95.0% CI for Exp (B)	
			Lower	Upper			Lower	Upper
Gender	0.010 *	1.598	1.117	2.286				
Age	0.530	1.118	0.789	1.585				
Tumor location	0.550	1.063	0.869	1.301				
Tumor size	0.014 *	1.578	1.098	2.267				
Histologic type	0.415	1.071	0.909	1.262				
Bornmann classification	1.95 × 10^−4^ ***	1.683	1.277	2.220	0.006 **	1.775	1.181	2.667
Differentiation	0.580	1.112	0.764	1.618				
Depth of invasion	1.07 × 10^−5^ ***	1.601	1.291	1.985				
Lymph-node metastasis	9.59 × 10^−9^ ***	1.658	1.390	1.978				
AJCC pTNM	5.54 × 10^−14^ ***	1.680	1.258	2.245				
IGF2BP2 expression	3.14 × 10^−6^ ***	2.323	1.618	3.333	0.003 **	2.009	1.268	3.184

* *p* < 0.05, ** *p* < 0.01, *** *p* < 0.001.

## Data Availability

The data contained in this research are available if requested. The publicly available data used in this research were acquired from the following databases: TCGA database (https://portal.gdc.cancer.gov/ (accessed on 20 September 2019)), Kaplan–Meier plotter online (http://www.kmplot.com/ (accessed on 20 September 2019)), and LinkedOmics (http://www.linkedomics.org/ (accessed on 26 November 2019)).

## Supplementary Materials

The following supporting information can be downloaded at: https://www.mdpi.com/article/10.3390/cancers14215381/s1, Figure S1: Representative results of the IGF2BP2 protein upregulation in GC specimens determined by western blotting; Figure S2: The protein level of IGF2BP2 in GC cell lines; Figure S3: Western blotting was performed to confirm the stable IGF2BP2-overexpressing AGS cells were constructed successfully; Figure S4: Western blotting was performed to confirm the stable IGF2BP2-overexpressing SGC7901 cells were constructed successfully; Figure S5: Western blotting was performed to confirm the stable IGF2BP2 knockdown MKN28 cells were constructed successfull; Figure S6: The protein levels of HMGA1 was up-regulated by IGF2BP2 overexpression in AGS cells; Figure S7: The protein levels of HMGA1 was up-regulated by IGF2BP2 overexpression in SGC7901 cells; Figure S8: The protein levels of HMGA1 was down-regulated by knocking down of IGF2BP2 in MKN28 cells; Figure S9: HMGA1 overexpression in MKN28 cells, in which IGF2BP2 knockdown was confirmed by Western blotting analysis; Figure S10: The amounts of HMGA1, E-cadherin, N-cadherin, ZEB1, Vimentin, and SNAI1 and in AGS, SGC7901, and MKN28 cells in which IGF2BP2 was overexpressed or knocked down were evaluated by Western blotting analyses.
