# Supplementary material for: IGF2BP2 Promotes Epithelial to Mesenchymal Transition and Metastasis through Stabilizing HMGA1 mRNA in Gastric Cancer
Source: Cancers (Basel). 2022 Oct 31;14(21):5381. doi: 10.3390/cancers14215381 (PMC9654580; doi:10.3390/cancers14215381)
Supplement: Supplementary file 1 [file cancers-14-05381-s001.zip › cancers-2002760-supplementary.pdf]

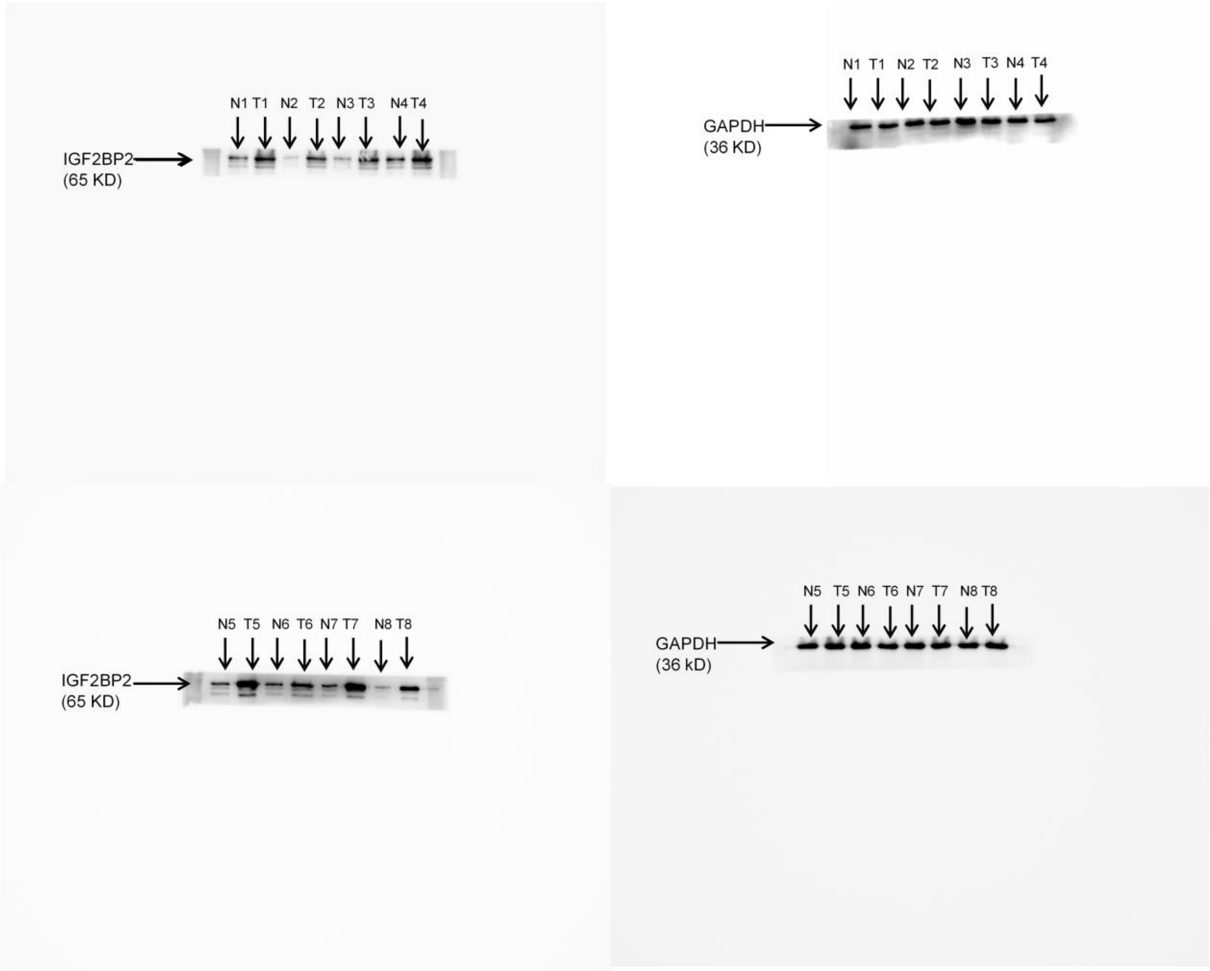

Figure S1. Representative results of the IGF2BP2 protein upregulation in GC specimens determined by western blotting.

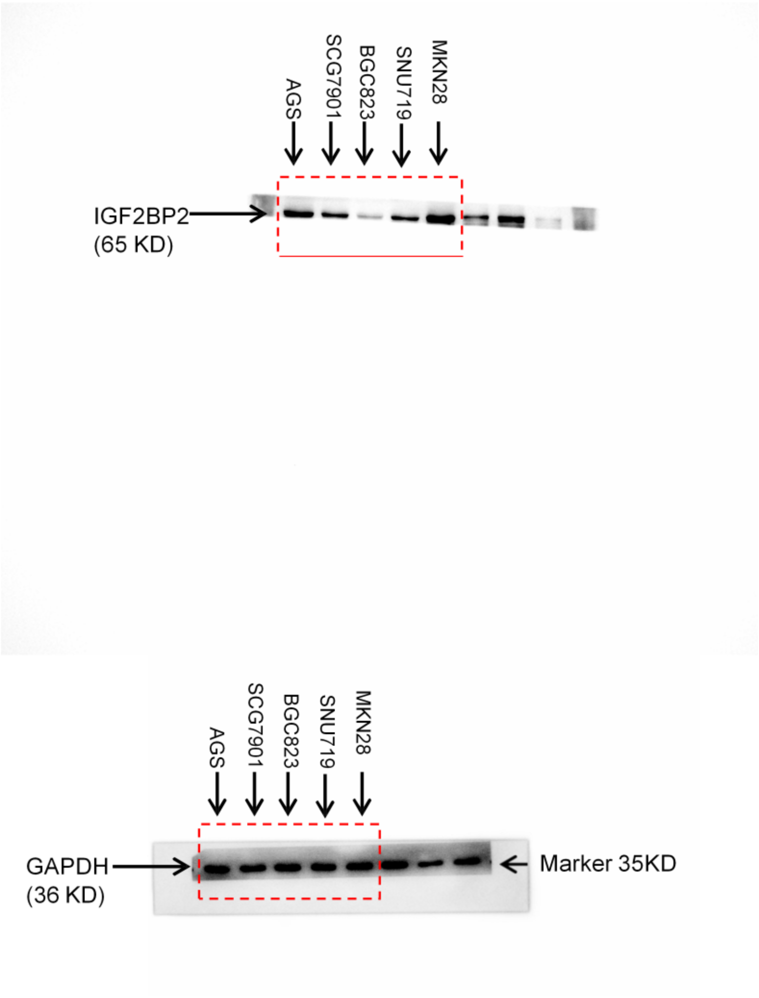

Figure S2. The protein level of IGF2BP2 in GC cell lines.

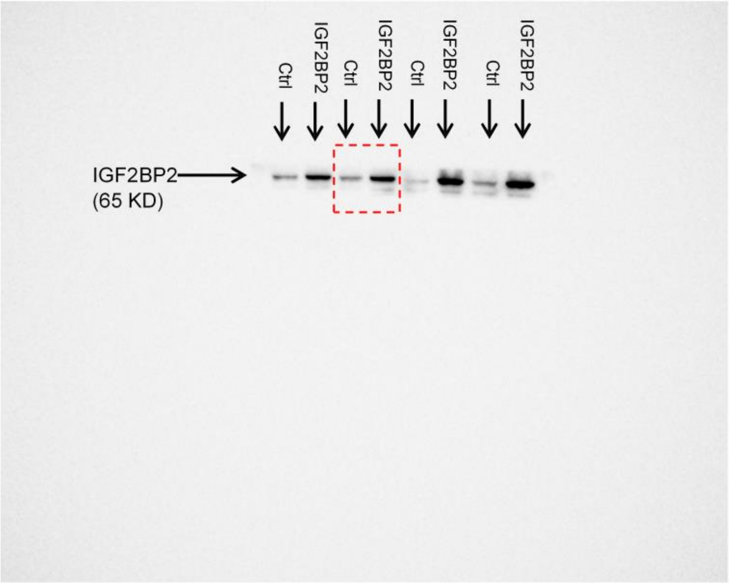

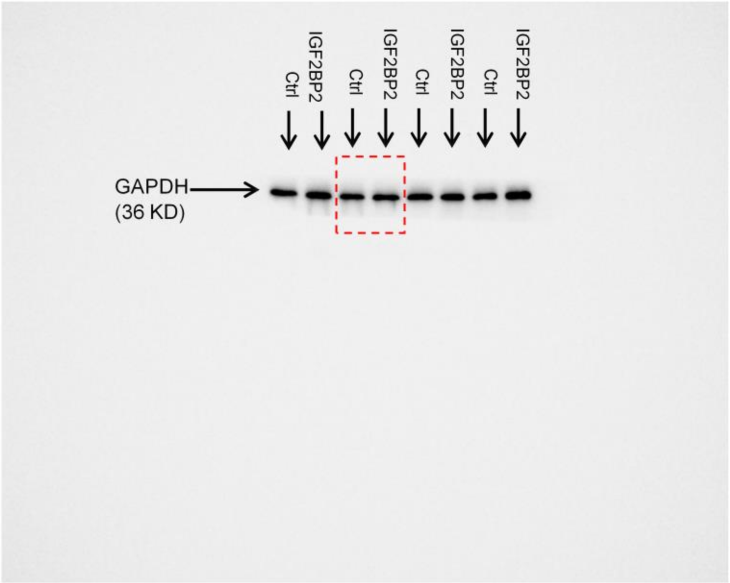

Figure S3. Western blotting was performed to confirm the stable IGF2BP2-overexpressing AGS cells were constructed successfully.

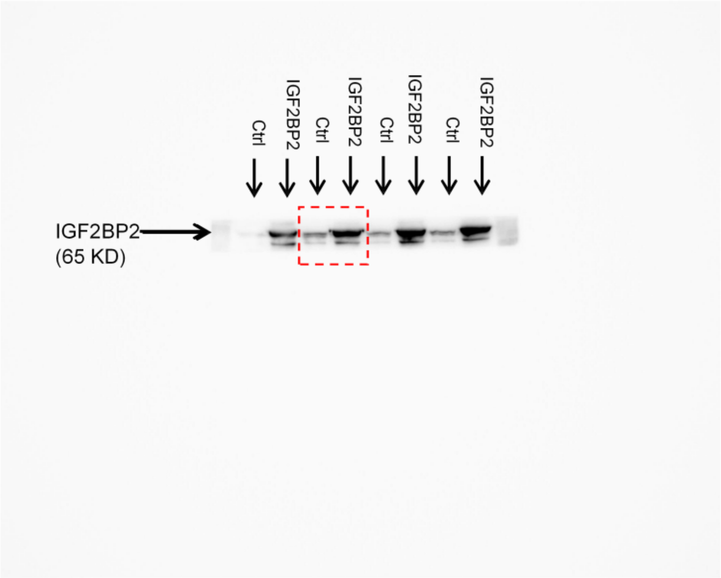

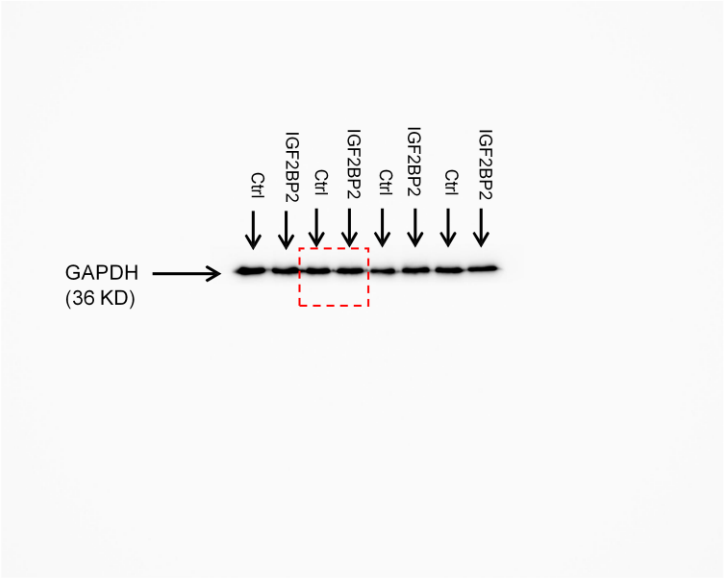

Figure S4. Western blotting was performed to confirm the stable IGF2BP2-overexpressing SGC7901 cells were constructed successfully.

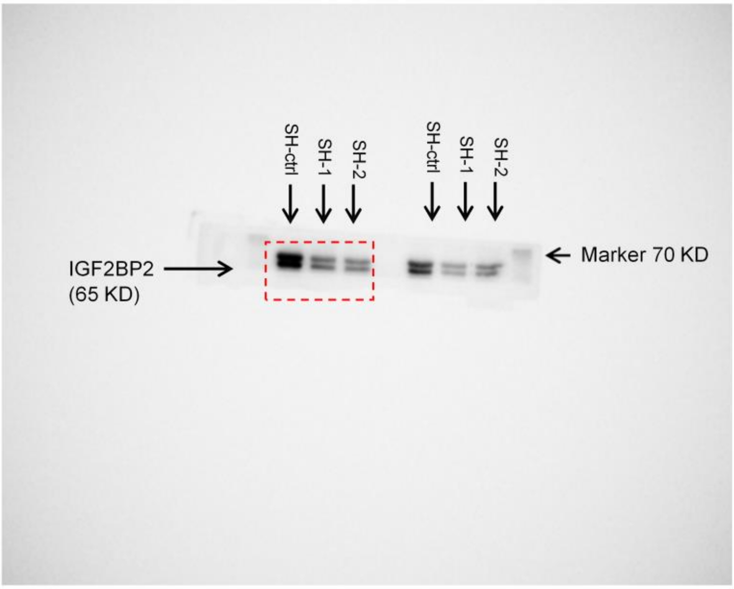

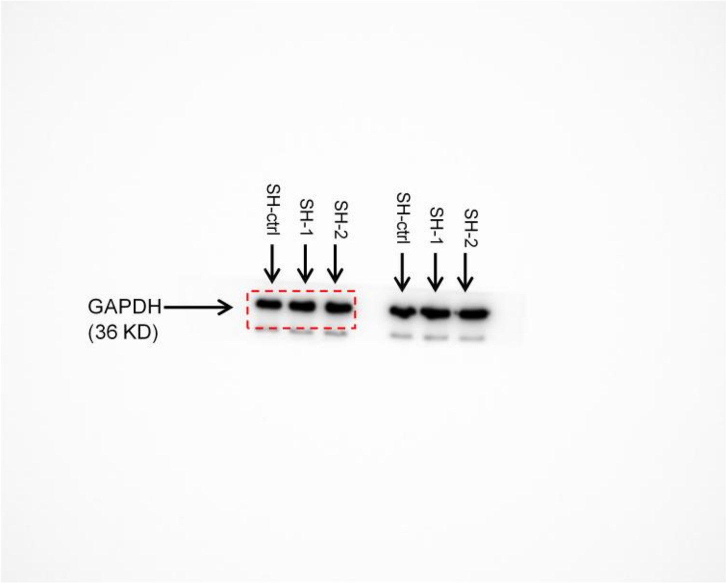

Figure S5. Western blotting was performed to confirm the stable IGF2BP2 knockdown MKN28 cells were constructed successfully.

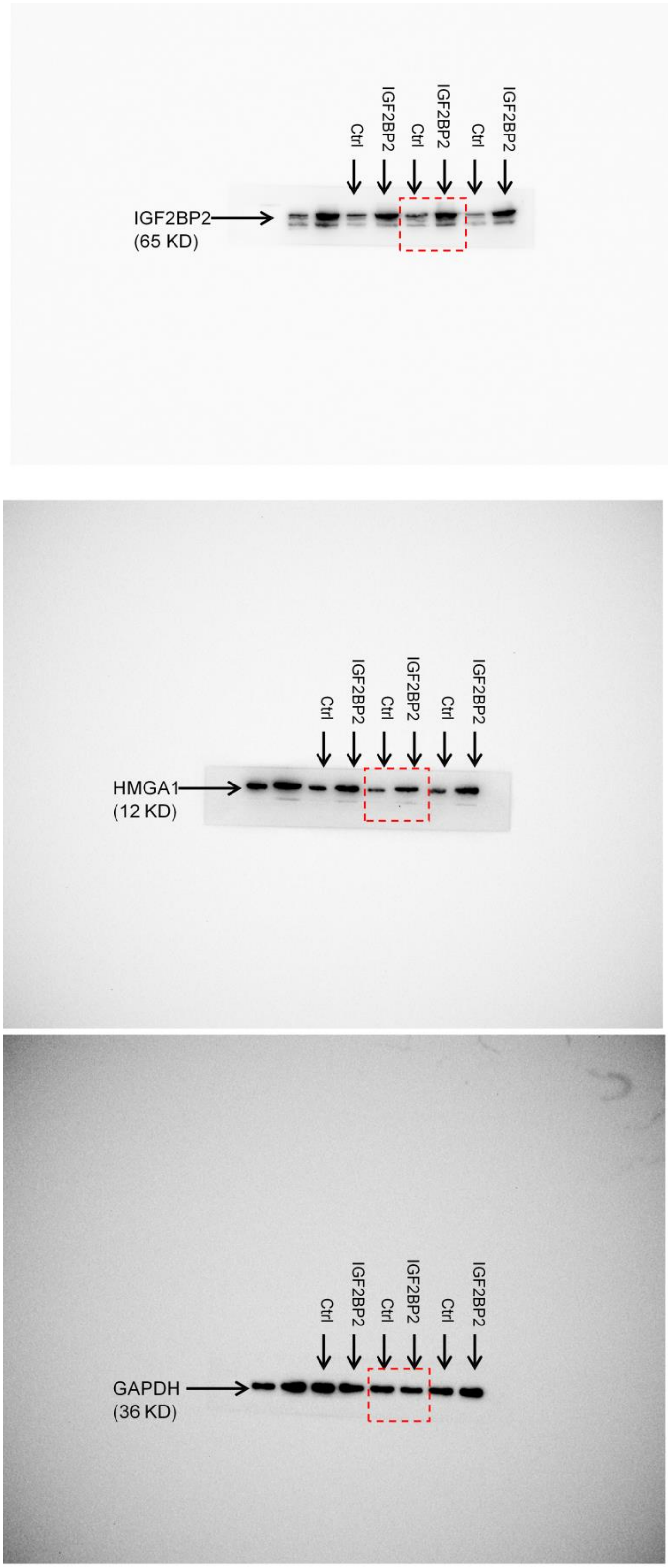

Figure S6. The protein level of HMGA1 was up-regulated by IGF2BP2 overexpression in AGS cells.

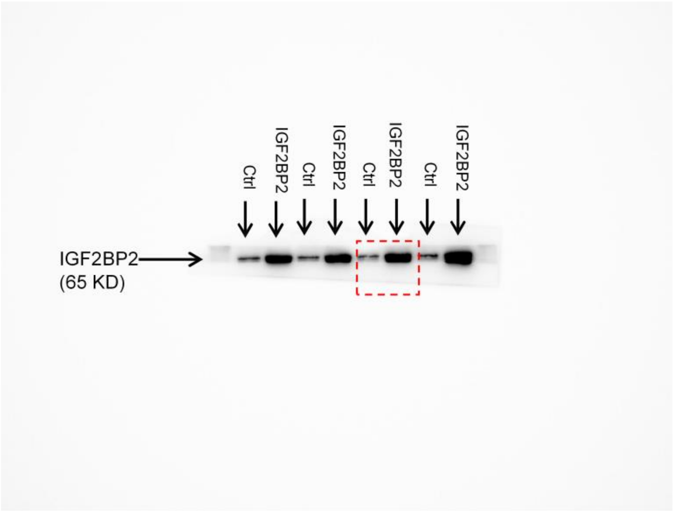

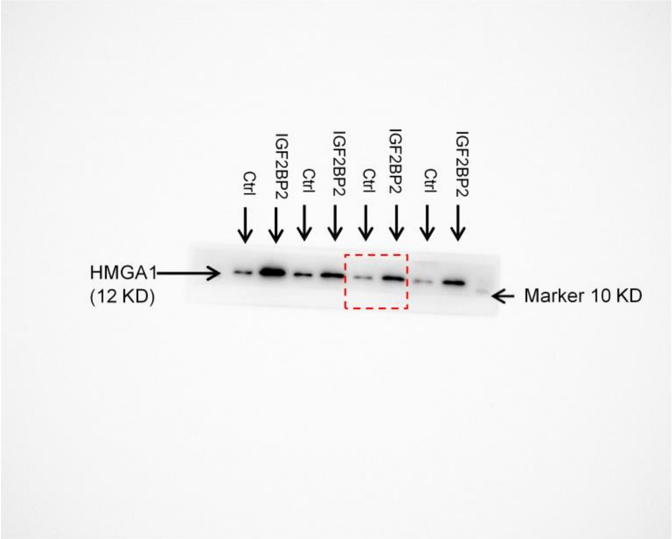

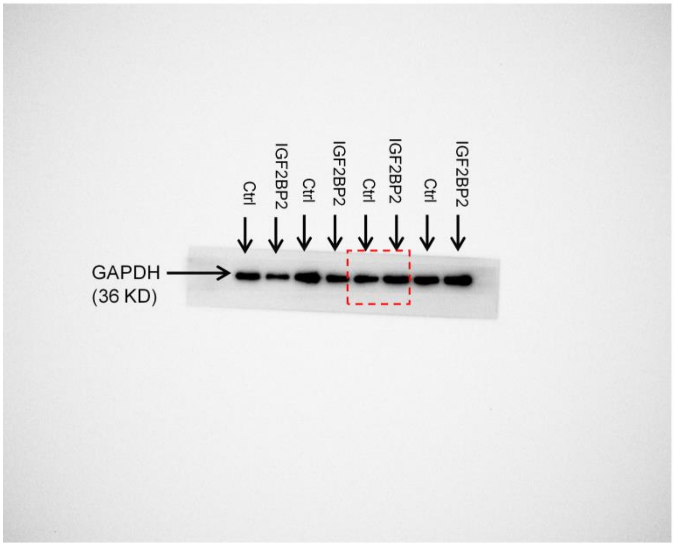

Figure S7. The protein level of HMGA1 was up-regulated by IGF2BP2 overexpression in SGC7901 cells.

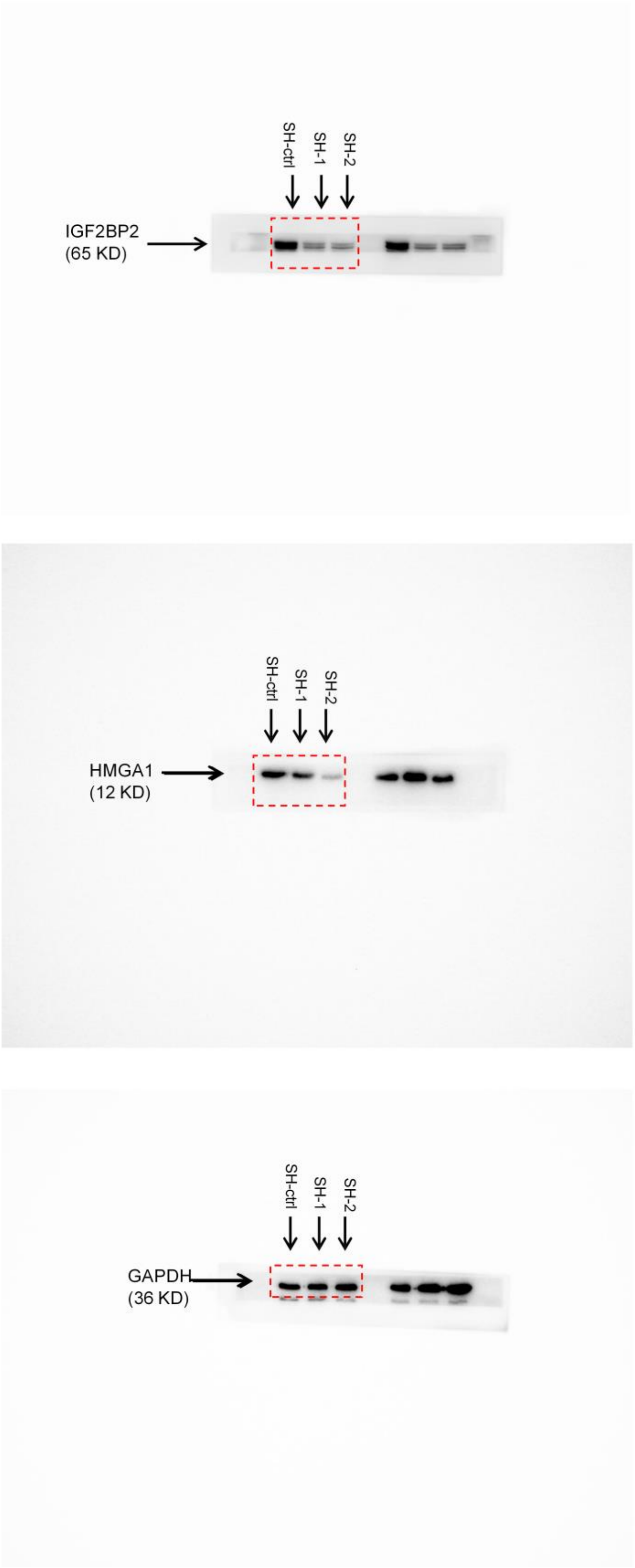

Figure S8. The protein level of HMGA1 was down-regulated by knocking down of IGF2BP2 in MKN28 cells.

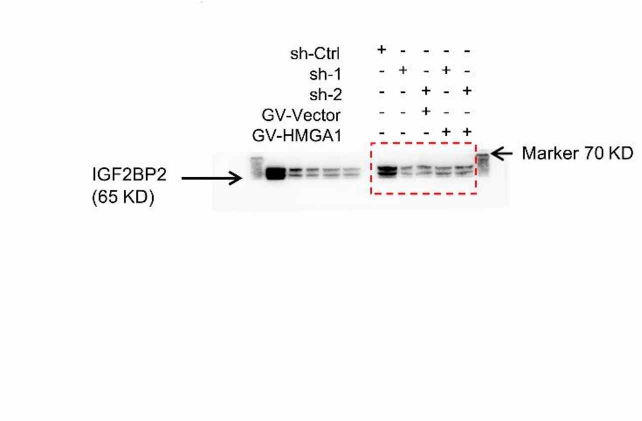

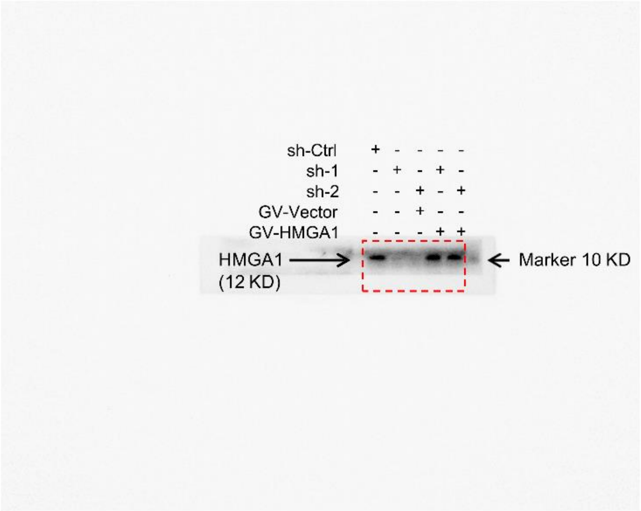

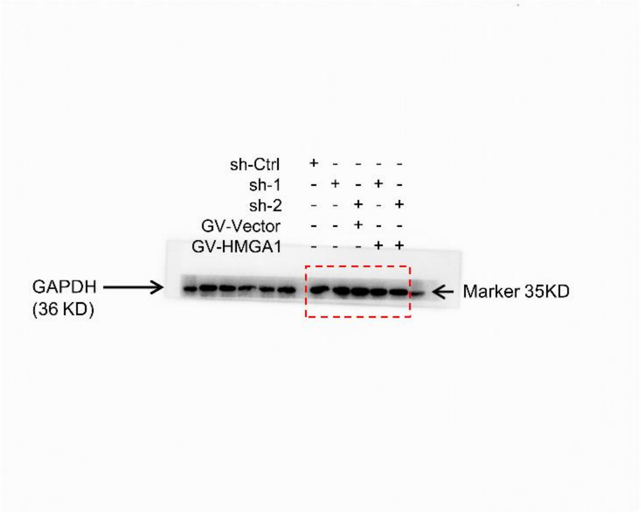

Figure S9. HMGA1 overexpression in MKN28 cells, in which IGF2BP2 knockdown was confirmed by Western blotting analysis.

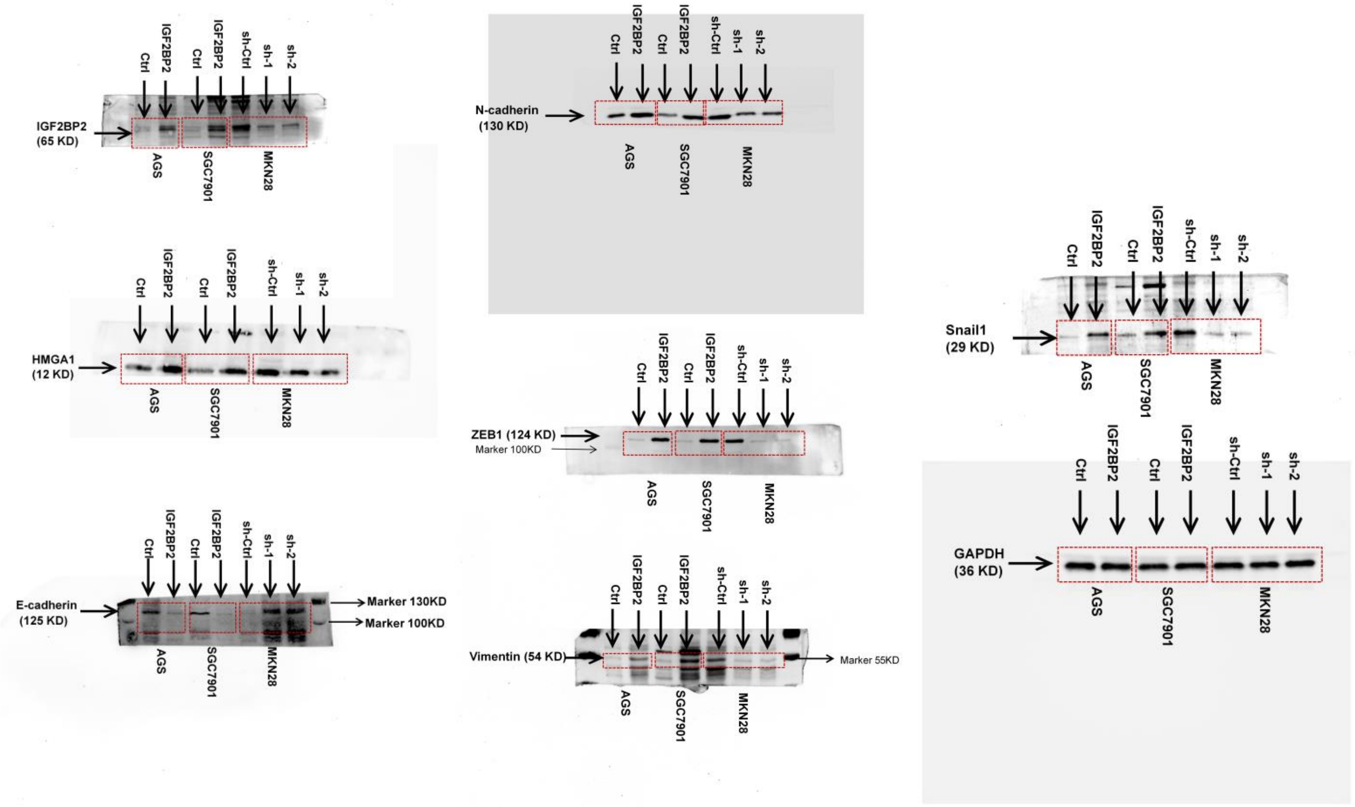

Figure S10. The amounts of HMGA1, E-cadherin, N-cadherin, ZEB1, Vimentin, and SNAI1 and in AGS, SGC7901, and MKN28 cells in which IGF2BP2 was overexpressed or knocked down were evaluated by Western blotting analyses.
